# Placental Vascular Malperfusion, Perinatal Death and Neonatal Brain Injury: A Mechanism-Based Narrative Review with Medico-Legal Implications

**DOI:** 10.3390/jcm15072734

**Published:** 2026-04-04

**Authors:** Helenia Mastrangelo, Matteo Antonio Sacco, Saverio Gualtieri, Gioele Grimaldi, Maria Daniela Monterossi, Giuseppe Neri, Isabella Aquila

**Affiliations:** 1 Department of Medical and Surgical Sciences, Institute of Legal Medicine, “Magna Graecia” University of Catanzaro, 88100 Catanzaro, Italymatteosacco@unicz.it (M.A.S.); saveriogualtieri@icloud.com (S.G.); gioele.grimaldi@studenti.unicz.it (G.G.); mariadaniela.monterossi@studenti.unicz.it (M.D.M.); 2 Anaesthesia and Intensive Care, Department of Medical and Surgical Sciences, “Magna Graecia” University of Catanzaro, 88100 Catanzaro, Italy; giuseppeneri91@gmail.com

**Keywords:** placental vascular malperfusion, maternal vascular malperfusion, fetal vascular malperfusion, stillbirth, fetal growth restriction, neonatal encephalopathy, angiogenic biomarkers, Doppler velocimetry, medico-legal assessment

## Abstract

**Background/Objectives:** Placental vascular malperfusion, on both the maternal (MVM) and fetal (FVM) side, is a key mechanism linking hypertensive disorders of pregnancy, fetal growth restriction (FGR), stillbirth, preterm neonatal death and neonatal encephalopathy. Nevertheless, clinical use and medico-legal interpretation of placental findings remain inconsistent. To summarize recent evidence on the relationship between placental vascular malperfusion, perinatal mortality and neonatal brain injury, integrating standardized placental pathology with Doppler and angiogenic biomarkers, and to outline the main medico-legal implications. **Methods:** A PubMed search using the string “((placenta OR placental pathology) AND (stillbirth OR fetal death) AND (maternal vascular malperfusion OR fetal vascular malperfusion))” yielded 118 records. After excluding reviews, meta-analyses, case reports (except one illustrative SARS-CoV-2 placentitis case), non-human studies and papers without original histopathology, 33 studies were included: observational cohorts and case–control studies with standardized placental assessment, autopsy series, biomarker/Doppler cohorts, mechanistic work, one randomized trial protocol and a small number of focused clinical commentaries. **Results:** Across these studies, MVM emerges as the dominant placental lesion in pre-eclampsia, FGR and a large proportion of stillbirths, especially in early-onset disease and in association with maternal hypertension. FVM is strongly linked to stillbirth and term neonatal encephalopathy, and specific combinations of MVM, FVM and inflammatory lesions correspond to distinct patterns of brain injury. Large population-based cohorts confirm that maternal hypertensive disorders and placental malperfusion are major upstream causes of intrauterine hypoxia and preterm neonatal death. Doppler velocimetry and angiogenic biomarkers (PlGF, sFlt-1 and their ratio) are strongly associated with an increased likelihood of underlying MVM and adverse neonatal outcomes, although their predictive performance remains probabilistic and context-dependent rather than diagnostic. Mechanistic studies suggest roles for placental genomic instability and altered decidual immunity in defective placentation. **Conclusions:** Maternal and fetal vascular malperfusion represent converging pathways to FGR, stillbirth, preterm neonatal death and neonatal encephalopathy. Routine, standardized placental examination, interpreted together with Doppler and biomarker data, substantially improves causal attribution and timing of injury, with direct consequences for counselling, prevention and medico-legal assessment.

## 1. Introduction

Perinatal mortality and severe neonatal morbidity remain major challenges worldwide, with South Asia alone accounting for more than one-third of stillbirths and a large share of preterm neonatal deaths [1,2]. Increasing evidence places the placenta at the centre of this burden: maternal vascular malperfusion (MVM) and fetal vascular malperfusion (FVM) represent key anatomic–pathologic pathways linking maternal disease, uteroplacental dysfunction and fetal/neonatal compromise [1,2,3,4,5,6,7].

The Amsterdam Placental Workshop Group Consensus has provided a standardized framework for classifying MVM, FVM and inflammatory lesions, which has been applied in multiple contemporary studies [1,2,3,6,7,8,9,10,11,12,13,14,15]. In parallel, longitudinal work on angiogenic biomarkers (PlGF, sFlt-1) and Doppler velocimetry has shown that abnormal angiogenic balance and increased uterine/umbilical vascular resistance reflect and anticipate placental malperfusion [4,9,11,16,17,18].

At the same time, medico-legal disputes around stillbirth and neonatal encephalopathy increasingly require robust, mechanism-based causal reconstruction, in which placental pathology, imaging and biomarkers must be integrated rather than considered in isolation. This narrative review synthesizes recent evidence on placental vascular malperfusion, perinatal death and neonatal brain injury, and discusses the implications for clinical practice and medico-legal evaluation.

Despite major progress in obstetric surveillance and neonatal intensive care, the global burden of placental-mediated morbidity and mortality remains largely unchanged, underscoring the need for deeper mechanistic understanding. A growing body of literature demonstrates that both MVM and FVM are not isolated histologic findings but the final expression of upstream biological failures, including defective trophoblast invasion, aberrant maternal–fetal immune interactions, endothelial dysfunction, and altered regulation of placental angiogenic pathways [9,17,18,19,20]. These processes often begin early in gestation and evolve subclinically for weeks or months before fetal decompensation becomes clinically apparent.

The recognition of MVM and FVM as distinct but frequently overlapping mechanistic pathways has prompted a shift from traditional symptom-based obstetric taxonomies (e.g., “pre-eclampsia”, “FGR”, “stillbirth”) toward mechanism-based disease models. Large cohorts such as PURPOSe [1,2] and analytic approaches including machine learning [21] reveal that syndromes historically treated as separate entities share a common placental architecture of chronic malperfusion, modulated by maternal cardiovascular status, metabolic milieu, thrombophilic predispositions and infectious/inflammatory triggers [2,7,14,15,19,22,23].

In this context, the placenta becomes not merely a post hoc diagnostic specimen but a living record of the intrauterine environment, encoding the sequence, chronicity and intensity of pathogenic events. MVM lesions such as distal villous hypoplasia, infarction and decidual arteriopathy indicate longstanding impairment of uteroplacental blood flow, whereas FVM patterns—including avascular villi and fetal vascular thrombosis—reflect disturbances in fetal perfusion that may coexist with, or be independent from, maternal vascular pathology. Importantly, recent evidence shows that these lesion patterns correlate with distinct fetal and neonatal phenotypes, particularly specific topographies of hypoxic–ischemic or inflammatory brain injury [13,24].

Moreover, the incorporation of angiogenic biomarkers and Doppler trajectories into clinical evaluation provides real-time insight into the evolving placental dysfunction. Abnormal PlGF and sFlt-1 levels often precede detectable structural disease, offering critical windows of opportunity for intensified monitoring or intervention [9,11,16,17]. These biochemical and biophysical signatures, when paired with standardized placental pathology, substantially strengthen causal inferences regarding the timing, mechanism and preventability of injury—key issues in medico-legal settings.

Finally, in light of the increasing complexity of maternal comorbidities and emerging infections (e.g., SARS-CoV-2) that interact with placental vascular function [14,15,23,25], understanding malperfusion has become indispensable for both clinical management and forensic investigation. A mechanism-based approach improves diagnostic accuracy, guides personalized surveillance strategies, and clarifies which adverse outcomes stem from chronic antepartum pathology versus acute intrapartum events.

## 2. Materials and Methods

A literature search was performed in PubMed using the Boolean string: ((placenta OR placental pathology) AND (stillbirth OR fetal death) AND (maternal vascular malperfusion OR fetal vascular malperfusion)).

No date or language restriction was applied at the search stage. The search yielded 118 records. Inclusion criteria were: Human studies with original placental histopathology including MVM and/or FVM (Amsterdam criteria or equivalent) [1,2,3,5,6,7,8,9,10,11,12,13,14,15,16,17,19,20,21,22,23,24,26,27,28]. Outcomes including at least one of the following: stillbirth, intrauterine fetal demise (IUFD), preterm neonatal death, FGR/SGA, pre-eclampsia or neonatal encephalopathy [1,2,3,5,6,7,9,11,12,13,14,15,16,17,21,22,23,24,26,27,28]. Study designs providing analyzable data: prospective or retrospective cohorts, case–control studies, autopsy series, biomarker/Doppler cohorts, mechanistic translational work, clinical trial or protocol, or targeted commentaries/clinical frameworks [1,2,3,5,6,7,8,9,10,11,12,13,14,15,16,17,18,19,20,21,22,23,24,25,26,27,28,29,30,31]. Exclusion criteria were: narrative reviews and meta-analyses (except one targeted review on post-pregnancy work-up [31]), case reports (except a single illustrative SARS-CoV-2 placentitis case [25]), non-human studies and papers lacking defined placental lesions.

**Figure 1:**
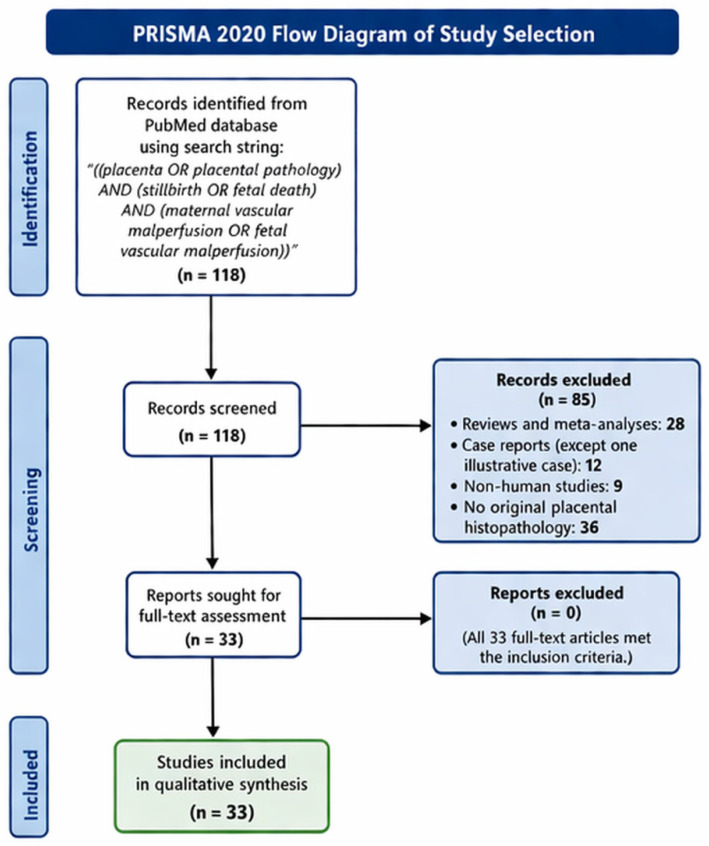
PRISMA flow diagram of study selection.

Titles and abstracts were screened, followed by full-text assessment. After exclusions, 33 studies were included [1,2,3,4,5,6,7,8,9,10,11,12,13,14,15,16,17,18,19,20,21,22,23,24,25,26,27,28,29,30,31,32,33]. The study selection process is illustrated in Figure 1. Most were observational cohorts or case–control/autopsy series with standardized placental assessment [1,2,3,5,6,7,8,9,10,11,12,13,14,15,17,21,22,24,26,27,28]; a minority explored biomarkers and Doppler [4,9,11,16,17,18], mechanistic or genomic aspects [19,20], or interventional strategies [16,30]. Three contributions were commentaries or clinical frameworks with direct relevance for interpretation and follow-up [29,31,33], and one was a detailed case report of SARS-CoV-2 placentitis with severe neonatal brain injury [25].

Data extracted included study design, population, gestational age range, definitions of placental lesions, principal maternal/placental/fetal outcomes and key effect estimates (RR, OR, aOR) where available.

### Definition of Placental Lesions

Maternal vascular malperfusion (MVM) and fetal vascular malperfusion (FVM) were defined according to the Amsterdam Placental Workshop Group Consensus criteria.

MVM lesions include features of impaired maternal uteroplacental perfusion, such as distal villous hypoplasia, accelerated villous maturation, infarctions, and decidual arteriopathy, reflecting defective spiral artery remodelling and chronic uteroplacental insufficiency.

FVM lesions comprise abnormalities of the fetal circulation within the placenta, including fetal vascular thrombosis, avascular villi, and villous stromal-vascular karyorrhexis, indicative of obstruction or stasis in the fetal vascular tree.

This review was conceived as a structured narrative synthesis rather than a formal systematic review or meta-analysis. Although a predefined search string and explicit inclusion/exclusion criteria were applied to enhance methodological transparency, no formal risk-of-bias assessment was performed and the review protocol was not prospectively registered. Given the heterogeneity of study designs (histopathology cohorts, biomarker studies, neuropathological analyses, mechanistic and translational research), quantitative pooling was not feasible. Therefore, the synthesis remains interpretative and potentially subject to selection bias, which should be considered when interpreting the conclusions.

## 3. Results

### 3.1. Placental Vascular Malperfusion: Spectrum and Standardization

Application of Amsterdam criteria reveals a broad spectrum of FVM and MVM lesions. In a series of 378 placentas with segmental FVM (SFVM), Stanek distinguished recent, established and remote lesions based on endothelial fragmentation, villous hypovascularity, avascular villi and segmental villous mineralization [8]. These “ages” differed in gestational age and clinical context (macerated stillbirth, mode of delivery) but not in underlying maternal or fetal conditions, suggesting that they represent temporal stages of a common process rather than different diseases [8]. Coexistence of lesions of different ages within a single placenta indicates repeated or ongoing fetal vascular insults—an important point when estimating timing of injury in forensic settings.

Using machine learning on 60 histologic variables in the Safe Passage Study, Petersen et al. identified distinct placental clusters dominated by severe MVM, FVM, other vascular lesions, inflammatory patterns or near-normal findings [21]. A severe MVM cluster was associated with a roughly 12-fold increase in stillbirth and nearly doubled preterm birth risk compared to the “normal” cluster, whereas the FVM cluster was particularly associated with SGA and reduced head circumference [21]. This supports a syndromic view of placental disease, in which combinations of lesions rather than single features drive risk.

### 3.2. Maternal Vascular Malperfusion, FGR and Perinatal Death

Across settings, MVM is consistently the dominant placental lesion in pre-eclampsia, FGR and stillbirth.

In a large Indian–Pakistani cohort of stillbirths (PURPOSe), maternal and fetal vascular malperfusion were the primary placental causes in 47% of cases, while intrauterine hypoxia was the primary fetal cause in 72% and hypertensive disease (mainly pre-eclampsia) the leading maternal cause (36%) [1]. Approximately 19% of stillbirths shared intrauterine hypoxia, placental malperfusion and pre-eclampsia/eclampsia as co-primary causes, highlighting a maternal–placental–fetal continuum [1].

In a companion PURPOSe analysis of preterm neonatal deaths, MVM/FVM were primary placental causes in 28% and intrauterine hypoxia or infection accounted for most neonatal deaths, again on a background of maternal hypertensive disease and placental pathology [2].

Kulkarni et al. [3] studied 1633 placentas from stillbirths, preterm neonatal deaths and term live births: MVM was found in 58.4% of stillbirths, 31.1% of preterm neonatal deaths and 15.4% of term live births [3]. Adjusted relative risks were ~3.9 for stillbirth and ~2.1 for preterm neonatal death vs. term live birth; FVM was also more frequent in stillbirths and conferred an adjusted risk of ~4.1 [3]. MVM was strongly associated with maternal hypertension, SGA and antepartum hemorrhage [3]. These risk estimates derive from specific study populations and analytical models, and their generalizability may vary according to geographic setting, referral patterns, baseline obstetric risk, and the degree of adjustment for confounding variables. Therefore, caution is warranted when extrapolating these associations to broader or different clinical contexts.

Within the PURPOSe network, Yogeshkumar et al. reported MVM in 57.3% of placentas from women with pre-eclampsia versus 37.1% in normotensive controls; histologically abnormal placentas were present in 61.3% vs. 45.0%, respectively [7]. MVM was particularly common when pre-eclampsia co-occurred with stillbirth or preterm neonatal death (≈80% of stillbirth + PE) [7]. In contrast, inflammatory lesions were less frequent with pre-eclampsia than in non-hypertensive pregnancies, reinforcing pre-eclampsia as the prototypical MVM-driven syndrome [7].

Case–control data from Italy demonstrate that elevated second-trimester uterine artery PI correlates with classical MVM lesions such as infarction, distal villous hypoplasia and retroplacental hematoma; higher UtA-PI quartiles carry increased odds of these lesions and of stillbirth [4]. Severe forms of pre-eclampsia are likewise characterized by more pronounced MVM and worse neonatal outcomes, with MVM independently associated with both severe maternal disease and adverse neonatal composite endpoints [26].

At term, a South African series of 89 singleton stillbirths showed significant associations between birthweight, placental weight, MVM and cord abnormalities, and suggested that systematic placental examination permits causal attribution in the majority of cases [28].

### 3.3. Fetal Vascular Malperfusion and Neonatal Brain Injury

While MVM underpins global placental insufficiency and FGR, FVM appears particularly relevant to neonatal encephalopathy and brain injury.

In Kulkarni’s study, FVM was significantly more frequent in stillbirths than in preterm neonatal deaths or term live births, with a four-fold increased risk of stillbirth [3].

Vik et al. [24] performed a case–control study of term/near-term neonatal encephalopathy (NE) and found global FVM in 20% of NE cases vs. 7% of controls, with a trend toward more segmental and high-grade fetal thrombotic vasculopathy in NE [24]. About one quarter of NE placentas showed some FVM, whereas MVM and inflammatory lesions did not differ substantially between cases and controls [24]. In NE with FVM, cardiotocography was more often abnormal, suggesting that chronic FVM may lower the threshold for intrapartum hypoxic injury [24].

Viaene et al. [13] combined standardized placental pathology with detailed neuropathology in 65 stillborn infants [13]. FVM was strongly associated with white matter injury, whereas amniotic fluid infection correlated with acute cortical and cerebellar neuronal injury and subarachnoid hemorrhage; high-grade chronic inflammation (e.g., VUE/CHI) was linked to hippocampal injury and MVM to acute neuronal injury in basal ganglia, brainstem and spinal cord [13]. This region-specific mapping supports the existence of distinct placento-cerebral injury pathways.

An extreme illustration is provided by SARS-CoV-2 placentitis: Benny et al. described two infants born to mothers infected in the second trimester who developed severe early-onset seizures, acquired microcephaly and progressive cystic encephalomalacia, in the absence of an obvious intrapartum sentinel event [25]. Placentas showed high SARS-CoV-2 load, FVM and intense inflammatory/oxidative signalling; in one infant, viral proteins were detected in the brain at autopsy [25]. Clinically, the picture mimicked hypoxic-ischaemic encephalopathy, but the underlying mechanism was a combination of chronic fetal vascular malperfusion and placental/fetal inflammation with probable in utero neuroinfection.

### 3.4. Infection, Inflammation and Interaction with Malperfusion

Acute (chorioamnionitis, funisitis) and chronic (VUE, CHI) inflammatory lesions often coexist with MVM and FVM and play a major role in preterm birth and fetal death.

In PURPOSe, placental infection and inflammation were frequent primary or contributing causes of stillbirth and preterm neonatal death, second only to MVM/FVM [1,2]. Manocha et al. found that in 100 IUFDs, MVM alone accounted for 30%, MVM + FVM for 10%, FVM alone for 6% and inflammatory lesions alone for 12%; only 18% remained unexplained [5]. In Kedar Sade’s series, placental lesions were the leading category of cause of death (28%), with MVM representing more than half of these, and ascending infection the second-most frequent category [6].

Bezemer et al. demonstrated that FGR, stillbirth and VUE are associated with a shift toward more pro-inflammatory decidual macrophages and altered T-cell/Treg profiles, suggesting that immune dysregulation at the maternal–fetal interface contributes to both malperfusion and chronic villitis [19].

COVID-19 pregnancy cohorts add another layer. Rebutini and Antolini-Tavares both observed higher frequencies of MVM (and to a lesser extent FVM and CHI) in infected women than in matched controls, particularly in symptomatic or severe cases, with increased preterm birth and infant death [14,15]. Morphometric changes were modest, and extensive placental destruction remained rare [14,15]. Rocha de Souza et al. [23] similarly reported MVM in 38% and inflammatory/infective lesions in ~9% of placentas from PCR-positive women, with more fetal deaths in cases with placental inflammation [23]. Overall, SARS-CoV-2 seems to amplify pre-existing placental vulnerability, with clinically relevant consequences in a subset of pregnancies.

Gonen et al. showed that early-onset placental abruption (<34 weeks) was associated with more severe MVM, maternal inflammatory response and worse neonatal neurologic outcome than late-onset abruption, which appeared more “acute” and less chronically diseased [22]. Thus, early abruption often represents the end-stage of chronic MVM/inflammation rather than a purely sudden event.

### 3.5. Doppler, Angiogenic Biomarkers and Prediction of Placental Disease

Several prospective cohorts have explored how Doppler and angiogenic biomarkers capture underlying placental pathology.

In FGR pregnancies, Hong et al. reported that MVM was the commonest placental lesion (≈51%), especially in early-onset FGR, and that MVM was strongly associated with low PlGF, elevated sFlt-1 and sFlt-1/PlGF ratio, as well as abnormal uterine and umbilical artery Doppler [9]. Severe non-neurological neonatal morbidity was highest in groups with MVM, FVM or CHI [9]. In a broader SGA/FGR cohort, abnormal CPR, high UA-PI, high mean UtA-PI, low PlGF and high sFlt-1/PlGF each independently increased the odds of any placental abnormality and of MVM [11].

Agrawal et al. linked serial PlGF and UtA Doppler patterns to specific histologic phenotypes [17]. MVM was characterized by progressively low PlGF and high UtA-PI; FVM and VUE typically had normal PlGF and Doppler; CHI and massive perivillous fibrinoid deposition showed very low PlGF despite mostly normal UtA-PI [17]. Among 29 stillbirths, 96.5% had at least one low PlGF before death, whereas UtA Doppler remained normal in most [17]. Thus, serial PlGF appears more sensitive than Doppler for identifying pregnancies destined to catastrophic placental failure, and particular PlGF/Doppler combinations help infer the type of placental disease.

Romero et al. proposed a mechanism-based “new taxonomy” of obstetrical syndromes by stratifying them according to presence or absence of MVM [18]. When MVM was present, differences in PlGF/sFlt-1 between cases and controls emerged earlier in gestation, and associations between abnormal ratios and pre-eclampsia, SGA, PPROM and PTL approximately doubled [18]. This supports the idea that biomarkers perform best when interpreted within a placenta-informed classification rather than against purely clinical definitions.

McLaughlin et al. constructed GA-specific PlGF reference curves and, in a small high-risk cohort with very low PlGF, suggested that adding LMWH to aspirin might partially restore PlGF levels, prolong gestation and reduce stillbirth in women with severe placenta-mediated disease; MVM was the predominant lesion at pathology [16].

Finally, Groten et al. designed a multicenter randomized trial testing pentaerithrityl tetranitrate in pregnancies with elevated UtA-PI as a strategy to treat uteroplacental malperfusion and reduce FGR and perinatal death [30].

### 3.6. Mechanistic Insights and Post-Pregnancy Work-Up

Huang et al. used placental RNA sequencing (data derived from a preprint publication and therefore pending peer review) to quantify genomic instability and hypoxia signatures, finding that higher instability was associated with earlier pre-eclampsia, MVM lesions and SGA, and that trophoblast stem cells from MVM + PE placentas showed more genomic instability and reduced extravillous trophoblast invasion [20]. This suggests a mechanistic link between placental genomic instability, defective invasion and MVM-driven disease.

De Moreuil et al. provided a practical framework for post-pregnancy work-up after a vasculo-placental disorder (VPD)—including severe pre-eclampsia, HELLP, IUGR, abruption and vascular stillbirth—emphasizing systematic placental review, targeted investigation for antiphospholipid syndrome and selected thrombophilias, and a combined internal medicine–obstetrics consultation around 2 months postpartum to plan secondary prevention and manage long-term cardiovascular risk (Table 1) [31].

## 4. Discussion

Across 33 studies, a coherent picture emerges:

MVM is the central placental lesion linking maternal hypertension, pre-eclampsia, FGR and a large proportion of stillbirths and preterm neonatal deaths [1,2,3,4,5,6,7,26,28].

FVM is a key determinant of stillbirth and neonatal encephalopathy, and of specific patterns of CNS injury, particularly white matter damage and increased vulnerability to intrapartum hypoxia [3,8,13,24,25].

Acute and chronic inflammatory lesions interact with vascular malperfusion and are especially relevant in preterm birth, infection-related losses and COVID-19 [1,2,5,6,14,15,19,22,23].

Angiogenic biomarkers and Doppler, when interpreted within a mechanism-based taxonomy, provide powerful tools to detect and phenotype placental disease before clinical decompensation [4,9,11,16,17,18].

Mechanistic work links decidual immune remodelling and placental genomic instability to defective placentation and MVM, offering future therapeutic targets [19,20].

Taken together, these findings support an integrated model of placental injury in which multiple upstream pathways—vascular, inflammatory, immunologic and genomic—converge to produce a spectrum of fetal and neonatal outcomes. Rather than functioning as isolated entities, MVM, FVM and inflammatory lesions appear to operate along interconnected axes that evolve dynamically during gestation. For example, chronic MVM in the setting of hypertensive disorders may predispose the fetus to growth restriction and hypoxia, while superimposed fetal vascular obstruction or inflammatory activation can modulate the severity and topography of injury, culminating in stillbirth or neonatal encephalopathy. This multilevel interaction explains why many cases of adverse perinatal outcome present clinically as heterogeneous syndromes, yet share remarkably consistent underlying placental mechanisms.

Similarly, the recurring observation that angiogenic biomarkers (such as PlGF and sFlt-1) and Doppler indices map closely onto MVM-driven disease underscores the importance of shifting from symptom-based to mechanism-based frameworks. These diagnostic tools not only predict which pregnancies harbour placental pathology but also capture its trajectory, offering a temporal window into the evolution of malperfusion. Their integration with histopathology improves causal inference by demonstrating whether the fetus was already compromised antenatally—an increasingly decisive element in both clinical decision-making and medico-legal evaluation.

Within this broader paradigm, structured post-VPD work-up, anchored in placental pathology and integrated internal medicine–obstetrics care, is both clinically rational and medico-legally relevant [31]. By identifying underlying predispositions such as thrombophilia, chronic vascular disease, abnormal immune responses or prior placental insufficiency, post-VPD pathways facilitate individualized prevention strategies for subsequent pregnancies while also establishing a clear etiologic framework that can be used to explain adverse outcomes to families and courts.

From a medico-legal perspective, standardized placental examination should be considered essential in stillbirth, severe pre-eclampsia, early-onset FGR, unexplained preterm neonatal death and significant neonatal encephalopathy. Integration of placental histopathology with biomarker and Doppler trajectories allows more accurate reconstruction of the timing and mechanism of injury, helping distinguish chronic antepartum disease from acute perinatal events. This distinction is central to clinical audit, counselling, and assessment of preventability. While placental pathology can substantially support causal reconstruction, it rarely allows precise dating of injury or definitive attribution of responsibility in isolation. Interpretation must therefore remain integrated, contextual, and probabilistic, taking into account clinical chronology, fetal monitoring data, biomarker trajectories, and alternative etiologies.

## 5. Conclusions

Future research should focus on biomarker-driven intervention trials targeting uteroplacental malperfusion, on validation of genomic and immunologic markers of placental disease, and on implementation of standardized post-VPD pathways across obstetric care systems. Beyond expanding scientific knowledge, such work will help operationalize mechanism-based obstetrics, refine prognostication, and provide more accurate frameworks for medico-legal reconstruction. In particular, integrating histopathology with longitudinal biomarker and imaging data represents a promising avenue toward truly personalized assessment of placental health and fetal risk.

## Figures and Tables

**Table 1:** Comprehensive table of the included study.

	Study (Author, Year)	Design/Sample Size	Primary Mechanistic Category	Placental Lesions Identified	Key Findings/Outcomes	Mechanistic & Medico-Legal Significance
1	McClure, 2022 [1]	PURPOSe stillbirth, n = 981	MVM/FVM	MVM/FVM 47%	Hypertension → placental insufficiency → hypoxia	Identifies maternal disease → placental pathway → fetal death.
2	Dhaded, 2024 [2]	PURPOSe neonates	MVM/FVM + infection	MVM/FVM primary placental causes in 28% of neonatal deaths	Shows continuum: hypertensive disorders → malperfusion → neonatal death.	
3	Kulkarni, 2021 [3]	Prospective, n = 1633	MVM + FVM	High-frequency MVM; FVM significant	MVM RR ≈ 3.9 for stillbirth; FVM RR ≈ 4.1	Establishes chronic malperfusion as dominant cause of fetal death; strong link with hypertension.
4	Amodeo, 2022 [4]	Case–control	MVM	Placental hypoplasia, infarction, DVH	High UtA-PI predicts classic MVM lesions	Doppler identifies chronic disease months before stillbirth.
5	Manocha, 2019 [5]	IUFD, n = 100	MVM/FVM/infection	MVM 30%, MVM + FVM 10%, infection 12%	Most deaths explainable	Highlights majority of IUFD as placental-driven, reducing “unexplained” cases.
6	Kedar Sade, 2025 [6]	Autopsy, n = 138	MVM + infection	MVM = 54% of placental causes	Holistic autopsy assigns cause in 72%	Strong medico-legal support for placental-driven death.
7	Yogeshkumar, 2023 [7]	PURPOSe PE, n = 3067	MVM	57% in PE; 80% in PE + stillbirth	MVM phenotype dominates PE	PE as quintessential MVM syndrome.
8	Stanek, 2021 [8]	Retrospective, n = 378	FVM timing	Segmental FVM (recent/established/remote)	Temporal staging correlates with clinical context	Enables forensic timing of fetal vascular injury; mixed-age lesions indicate repetitive antenatal insults, not a sudden intrapartum event.
9	Hong, 2024 [9]	Prospective FGR, n = 301	MVM	MVM, VUE, FVM, CHI	MVM predicts low PlGF, abnormal Doppler, neonatal morbidity	Supports FGR as placenta-mediated disease; biomarkers reconstruct antenatal deterioration.
10	Cersonsky, 2023 [10]	Case–control (SCRN)	MVM/FVM (macroscopic)	Macro-MVM, Macro-FVM	Both significantly higher in stillbirth	Gross exam remains highly informative in resource-limited or incomplete autopsy settings.
11	Hong, 2025 [11]	Prospective SGA/FGR, n = 367	MVM (biomarkers)	MVM, FVM	Abnormal CPR, UA Doppler, PlGF, sFlt-1/PlGF predict MVM	Reinforces biomarker-driven phenotyping of placental disease.
12	Brink, 2022 [12]	Cohort, n = 1101	Inflammation vs. MVM	SPTB: inflammation; IPTB: MVM/FVM	Two distinct PTB pathways	Distinguishes acute inflammatory PTB vs. chronic malperfusion.
13	Viaene, 2025 [13]	Stillbirth neuropathology, n = 65	MVM/FVM/infection	MVM → basal ganglia/brainstem injury; FVM → white matter injury; infection → cortical injury	Provides a map of lesion-specific CNS injury, crucial for timing and causation.	
14	Rebutini, 2021 [14]	COVID case–control	MVM/FVM + CHI	Increased MVM/FVM, CHI in symptomatic infection	Higher prematurity, neonatal death	Confirms infection as co-factor in malperfusion.
15	Antolini-Tavares, 2023 [15]	COVID, n = 91 placentas	MVM + CHI	MVM increased in early infections	Severe maternal COVID worsens MVM	Timing of infection crucial for pathology.
16	McLaughlin, 2022 [16]	Mixed cohort	MVM + biomarkers	MVM	LMWH increases PlGF & prolongs gestation	Shows biochemical modifiability of placental perfusion.
17	Agrawal, 2022 [17]	Retrospective, n = 337	Biomarker phenotyping	MVM, FVM, VUE, CHI	Serial PlGF identifies impending stillbirth even with normal Doppler	Biomarker trajectory strongly supports antepartum onset.
18	Romero, 2022 [18]	Retrospective, n = 1499	MVM (biomarker taxonomy)	MVM	Abnormal PlGF/sFlt-1 predicts outcomes only in MVM pregnancies	Biomarkers meaningful only when stratified by mechanism, not by clinical labels.
19	Bezemer, 2020 [19]	Immune profiling	Immune/inflammatory	VUE, macrophage/Treg shifts	Present in FGR and stillbirth	Demonstrates immune dysregulation as root of chronic placental disease.
20	Huang, 2025 [20]	RNA-seq mechanistic	Genomic instability → MVM	Higher instability → early PE, MVM, SGA	Provides molecular explanation for defective trophoblast invasion.	
21	Petersen, 2023 [21]	Machine learning	MVM/FVM clusters	MVM cluster, FVM cluster	Severe MVM cluster → 12-fold stillbirth risk	Placental disease is syndromic, not isolated features.
22	Gonen, 2021 [22]	Retrospective, n = 305	MVM + inflammation	Severe MVM, maternal inflammatory response	Early abruption = chronic disease, not acute	Forensically shifts causal pathway from “sudden” to longstanding pathology.
23	Rocha de Souza, 2024 [23]	COVID cohort, n = 226	MVM + infection	MVM, inflammatory changes	Fetal death linked to placental inflammation	Viral infection worsens pre-existing vascular disease.
24	Vik, 2018 [24]	Case–control NE	FVM	Global/segmental FVM	FVM frequent in NE; increased CTG abnormalities	Identifies FVM as precondition for hypoxic brain injury.
25	Benny, 2023 [25]	Case series (2 neonates)	FVM + inflammation	Severe FVM + SARS-CoV-2 placentitis	Severe early encephalopathy & cystic encephalomalacia	A model of chronic vascular + inflammatory placental disease mimicking HIE in absence of intrapartum sentinel events.
26	Weiner, 2018 [26]	PE severity	MVM	MVM associated with severe PE and neonatal morbidity	MVM severity mirrors disease severity and duration.	
27	Weiner, 2017 [27]	Twin vs. singleton PTB	Inflammation + MVM	Singleton PTB linked to MVM/inflammation	Identifies mechanistic differences by pregnancy type.	
28	Vilane, 2025 [28]	Term stillbirths, n = 89	MVM/FVM + infection	Placental exam clarifies cause in >90%	Practical demonstration of placental pathology’s diagnostic power.	
29	Carbillon, 2022 [29]	Commentary	MVM	—	MVM as unifying mechanism	Conceptual framework for mechanism-based diagnosis.
30	Groten, 2019 [30]	RCT protocol	Therapeutic targeting of MVM	PETN to improve uteroplacental perfusion	First attempt to treat malperfusion directly.	
31	de Moreuil, 2025 [31]	Clinical framework	Post-VPD evaluation	Structured postpartum work-up	Provides standardized medico-legal pathway for recurrence risk & prevention.	
32	Rodriguez-Sibaja, 2024 [32]	Retrospective	MVM	MVM	ISUOG/SMFM FGR criteria detect MVM but with poor individual precision	Shows need for integrated Doppler + biomarker approach.
33	Kulkarni et al., 2022 [33]	Commentary	MVM + FVM	—	Reinforces centrality of malperfusion	Emphasizes chronicity of lesions.

## Data Availability

No new data were created or analyzed in this study. Data sharing is not applicable to this article.
